# MGC: a metagenomic gene caller

**DOI:** 10.1186/1471-2105-14-S9-S6

**Published:** 2013-06-28

**Authors:** Achraf El Allali, John R Rose

**Affiliations:** 1 Department of Computer Science and Engineering, University of South Carolina, 315 Main Street. Columbia, SC 29208, USA

## Abstract

**Background:**

Computational gene finding algorithms have proven their robustness in identifying genes in complete genomes. However, metagenomic sequencing has presented new challenges due to the incomplete and fragmented nature of the data. During the last few years, attempts have been made to extract complete and incomplete open reading frames (ORFs) directly from short reads and identify the coding ORFs, bypassing other challenging tasks such as the assembly of the metagenome.

**Results:**

In this paper we introduce a metagenomics gene caller (MGC) which is an improvement over the state-of-the-art prediction algorithm Orphelia. Orphelia uses a two-stage machine learning approach and computes a model that classifies extracted ORFs from fragmented sequences. We hypothesise and demonstrate evidence that sequences need separate models based on their local GC-content in order to avoid the noise introduced to a single model computed with sequences from the entire GC spectrum. We have also added two amino-acid features based on the benefit of amino-acid usage shown in our previous research. Our algorithm is able to predict genes and translation initiation sites (TIS) more accurately than Orphelia which uses a single model.

**Conclusions:**

Learning separate models for several pre-defined GC-content regions as opposed to a single model approach improves the performance of the neural network as demonstrated by the experimental results presented in this paper. The inclusion of amino-acid usage features also helps improve the overall accuracy of our algorithm. MGC's improvement sets the ground for further investigation into the use of GC-content to separate data for training models in machine learning based gene finders.

## Background

In cultured microbes, the shotgun sequences that result from sequencing the full genome come from a single clone which makes the assembly and annotation of the genome manageable. In metagenomics, the uncultured microbes are sampled directly from their environment. Next generation sequencing (NGS) used in metagenomics results in a much larger amount of data than traditional sequencing. However, the resulting sequences are noisy, partial and most importantly, may come from thousands of different species. Therefore, the assembly and annotation of the large metagenomics data present more challenges. Several methods have shown promising results and efficiency in assembling metagenomic data [3,4]. However these methods are designed for single genomes. Consequently they don't work well in cases where there are multiple species present as is the case in environmental samples. One way to deal with these difficulties is to bypass assembly and go directly to finding genes.

New methods are being developed to predict genes specifically in metagenomics. The best known methods in this field are MetaGene [5], Orphelia [1], and FragGeneScan [7]. MetaGene uses a similar approach to GeneMark.hmm [6] which takes into account the GC-content sensitive monocodon and dicodon models computed from fully annotated genomes. Once MetaGene extracts all the possible open reading frames (ORFs) present in the fragments, it uses statistical models computed from fully annotated genomes to score the fragments. The next step uses a dynamic programing algorithm that combines the previous score with the ORF length, the distance between the ORF and its neighbor, and the distance between the translation initiation start (TIS) and the left-most start codon. The goal of the dynamic programing algorithm is to select the final set of ORFs by resolving the overlap between ORFs. The scoring system is based on the log-odds ratios of observed frequency in coding ORFs and observed frequency in random ORFs. Two models are used by MetaGene, one for bacteria and one for archaea. These are automatically selected based on the outcome of a pre-defined domain classification method during the classification. MetaGene has been tested on randomly sampled fragments of size 700 bp from 12 annotated whole genomes. The results show the ability of MetaGene to predict genes with high sensitivity and slightly lower specificity. Orphelia obtains better performance than MetaGene by using a two-stage machine learning approach. The first stage builds linear discriminants for monocodon and dicodon usage as well as the TIS features extracted from the ORFs. This step linearly extracts features from the high dimensional features obtained from the codon usage and the TIS information, reducing each usage to a single feature. The next stage combines the features obtained from the linear discriminants as well as length and GC-content features using a non-linear neural network which produces the probability that a given ORF encodes a protein. Finally, Orphelia deploys a post-processing algorithm which uses probabilities from its scoring scheme in order to resolve the overlap. Orphelia is tested in a similar way to MetaGene, however more extensive experiments have been conducted including studying the effect of different fragment lengths, the accuracy of the program in predicting the TIS as well as complete vs. incomplete prediction capability of the program.

FragGeneScan is an algorithm based on hidden Markov models (HMM) capable of predicting genes in both complete genomes and metagenomic fragments [7]. The algorithm combines codon usage, sequence patterns for start/stop codons and sequencing error models using HMMs. The Viterbi algorithm is used to decide the best path of hidden states that generates the observed nucleotide fragment. The accuracy of FragGeneScan in short reads was compared to that of MetaGene. For simulated 700 bp reads with no sequencing error, FragGeneScan and MetaGene achieve comparable performance [7]. However, for shorter reads and reads with sequencing errors, FragGeneScan shows consistently better performance over MetaGene [7].

In this paper we introduce a new metagenomics gene caller called MGC which is based on a two-stage machine learning approach similar to that of the state-of-the-art program Orphelia [1]. MGC learns separate models for several pre-defined GC ranges as opposed to the single model approach used by Orphelia and applies the appropriate model to each fragment based on its GC-content. Chan and Stolfo [8] investigated model combination for machine learning classification and showed that models learned from disjoint partitions of a dataset outperform a single model learned from the entire dataset. Separating the training data by GC-content provides MGC with mutually exclusive partitions of the data in order to train multiple models.

We use GC-content to partition the training dataset for our two-stage machine learning approach. The use of GC-content for this purpose is inspired by the causal relationship between nucleotide bias and amino acid composition. Singer and Hickey [9] demonstrated that nucleotide bias can have a dramatic effect on the amino acid composition of the encoded proteins, they showed that GC-poor genomes have proteins that are rich in the FYMINK amino acids and GC-rich genomes have proteins that are rich in the GARP amino acids. This effect is not only present in complete genomes but it is also valid for individual genes. Singer and Hickey [9] identified genes common between a GC-rich genome (*B. burgdorferi*) and a GC-poor genome (*M. tuberculosis*) and measured the synonymous nucleotide frequencies and amino acid contents of each gene. While there was no overlap in the synonymous GC-contents of these two genomes, some overlap in the amino acid proportions of the encoded proteins exists. However, no overlap in the amino acid proportions of the encoded proteins in the common genes was found, the GARP/FYMINK ratio in the *M. tuberculosis *homolog was higher than the ratio of the corresponding gene in *B. burgdorferi*. Separating the models by GC-content can ensure that both compositions are accounted for instead of combining them into one model.

GC-content influences codon usage which in turn influences the amino acid usage. Lightfield et al. [10] have shown that across bacterial Phyla, distantly-related genomes with similar genomic GC-content have similar patterns of amino acid usage. They examined codon usage patterns and were able to predict protein amino acid content as a function of genomic GC-content. Lightfield et al. [10] demonstrated that use of amino acids encoded by GC-rich codons increased by approximately 1% for each 10% increase in genomic GC-content, the opposite was also true for GC-poor codons. Separating GC-contents into several GC ranges will ensure that the different linear discriminants can separate the codon and amino acid usage more precisely.

Another effect of GC-content is its link to the length of the genes. GC-rich genes in prokaryotes tend to be the longest while GC-poor genes tend to be the shortest [11]. The longer the gene is, the more candidate TIS codons the ribosome encounters. Unlike the ribosome, models find it hard to pick the correct TIS from a large number of candidates especially when they are close to each other. In addition to the number of candidate TIS codons, these candidates share most of the TIS window used to compute the features. Having separate models for genes that have a large number of start codons will ensure that the subtle difference between the candidates is learned by the non-linear neural networks.

In addition to separating the models by GC-content, MGC uses two amino acid features motivated by the benefit that these features have demonstrated in our previous research [2]. The use of amino acid composition as a protein feature is an early discovery. Amino acid bias has been used in several identification problems such as gene expression [12], protein identification [13], family classification [14] and protein secondary structure prediction [15]. For example, Misawa and Kikuno have found that the effect of amino acid composition on gene expression is stronger than that of the codon composition [12]. In a survey of codon and amino acid frequency bias in microbial genomes, Merkl found that optimizing translational efficiency has an effect on biased amino acid composition [16]. If a cell requires certain proteins in large quantities then the amino acids that consumes less energy during translation appear more frequently [16]. This bias is not adequately represented by GC content or codon usage. We hypothesise that amino acid usage provides our models with species-specific differences caused by protein synthesis energy constraints.

## Methods

### Datasets

We use the same two datasets used by Orphelia, one for training the neural network models and a second one for testing the MGC algorithm. The first dataset consists of 131 fully sequenced Bacterial and Archael genomes and their corresponding gene annotations obtained from GenBank [17] and the second dataset is comprised of ten Bacterial and three Archael genomes. Hoff et al. [18] list all the genomes used for training the Orphelia neural network in the supplementary materials of their paper and all genomes used for testing in Table 1 of their publication. The n-fold coverage for a genome is defined as the amount of sampled DNA that is equal in total length to n-times as the length of the original genome complete sequence. Fragments of 700 bp are randomly excised to create a 1-fold genome coverage for each genome in the training dataset and a 5-fold coverage for each genome in the testing dataset.

Two additional training datasets (different from the neural network training data) are used for the preprocessing step required in feature extraction. The first dataset is used for preprocessing the codon usage as described in the next section. Sequences are randomly sampled to create a 0.5-fold genome coverage. Annotated genes serve as positive examples (≈ 1.9 × 10^5 ^examples) and the longest ORF in each non-coding ORF-set serve as the negative examples (≈ 2.8 × 10^6 ^examples). The second dataset is used to preprocess the TIS feature which will be described in the next section. Symmetric windows of 60 bp around the TIS of the previously selected genes in the first dataset serve as positive examples (≈ 1.9 × 10^5 ^examples) while similar windows around the remaining start codons forming the ORF-set of each gene serve as negative examples (≈ 5.6 × 10^6 ^examples).

**Figure 1:**
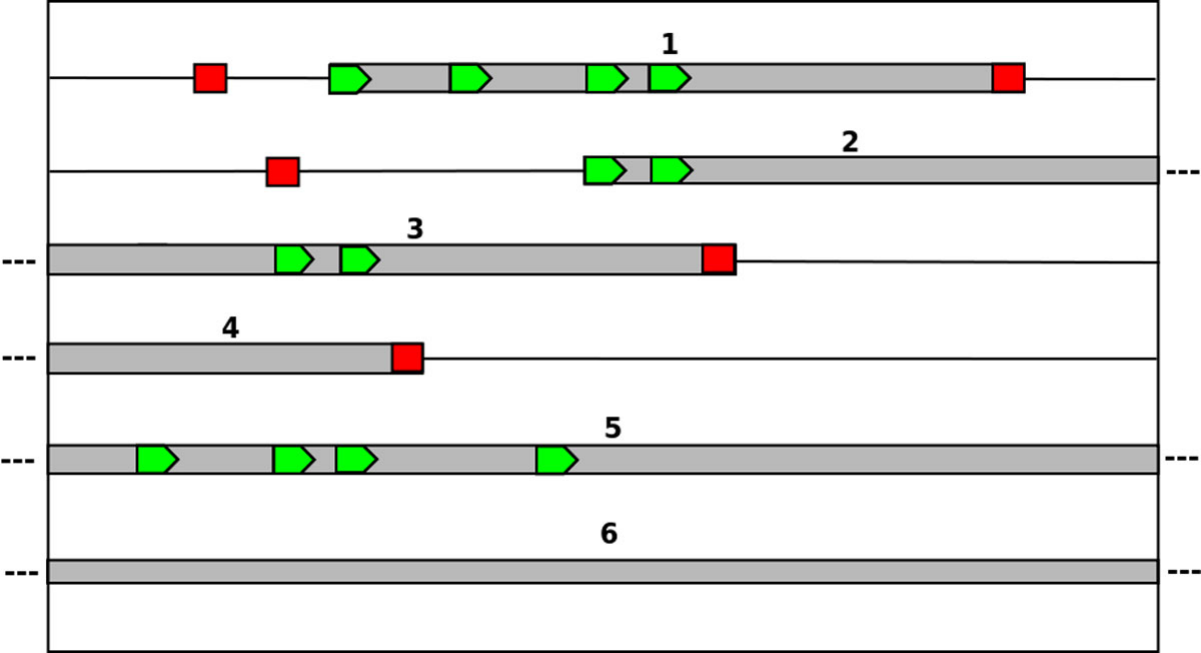
**Delineation of the possible ORF positions within the forward strand of a fragment**. The fragment is depicted by the outside box and gray bars represent possible ORFs. Candidate translation initiation sites are represented by green pentagons and red squares indicate stop codons.

ORFs are then extracted from all the fragments and divided into coding and non-coding ORFs based on the annotation of the genome. Two different types of ORFs are obtained. The ORFs that have both the start codon (either ATG, CTG, GTG or TTG) and the stop codon (either TAG, TGA or TAA) are referred to as complete ORFs. Incomplete ORFs are missing the upstream end, the downstream end or both in which case the ORF spans the entire fragment length without any start or stop codons being present. The addition of the incomplete ORFs notion is necessary since the ORFs present in fragments often stretch beyond these fragments making the standard ORF (complete ORFs) definition insufficient. In this paper, we refer to complete and incomplete ORFs simply as ORFs. Only ORFs with a minimal length of 60 bp are considered in both training and testing. Figure 1 illustrates the different locations of an ORF in a fragment. In addition to their DNA sequences, all the extracted ORF sequences are translated into the equivalent amino-acid sequences (for coding) and pseudo-amino-acid sequences (for non-coding).

In order to train the neural network, ORFs are extracted from the neural network training dataset and divided into positive and negative examples. ORFs from annotated genes serve as the positive examples (≈ 2.6 *× *10^6 ^examples) while one randomly selected ORF out of each non-coding ORF-set make up the negative examples (≈ 4.5 × 10^6 ^examples).

### The MGC algorithm

MGC is a metagenomic gene caller based on a two-stage machine learning approach similar to that of the state-of-the-art program Orphelia [1]. The first stage consists of linear discriminants that reduce a high dimensional feature space into a smaller one. For example, the linear discriminant for the dicodon usage reduces the 4096 dicodon frequencies into a single feature. However, these features are not linear across the entire GC spectrum. GC-content has a direct effect on codon and amino acid usages which means that fragments with similar GC-content should have similar features. Therefore, building different linear discriminants for each GC range will result in a better linear combination of the feature space which will better characterize the coding class.

**Figure 2:**
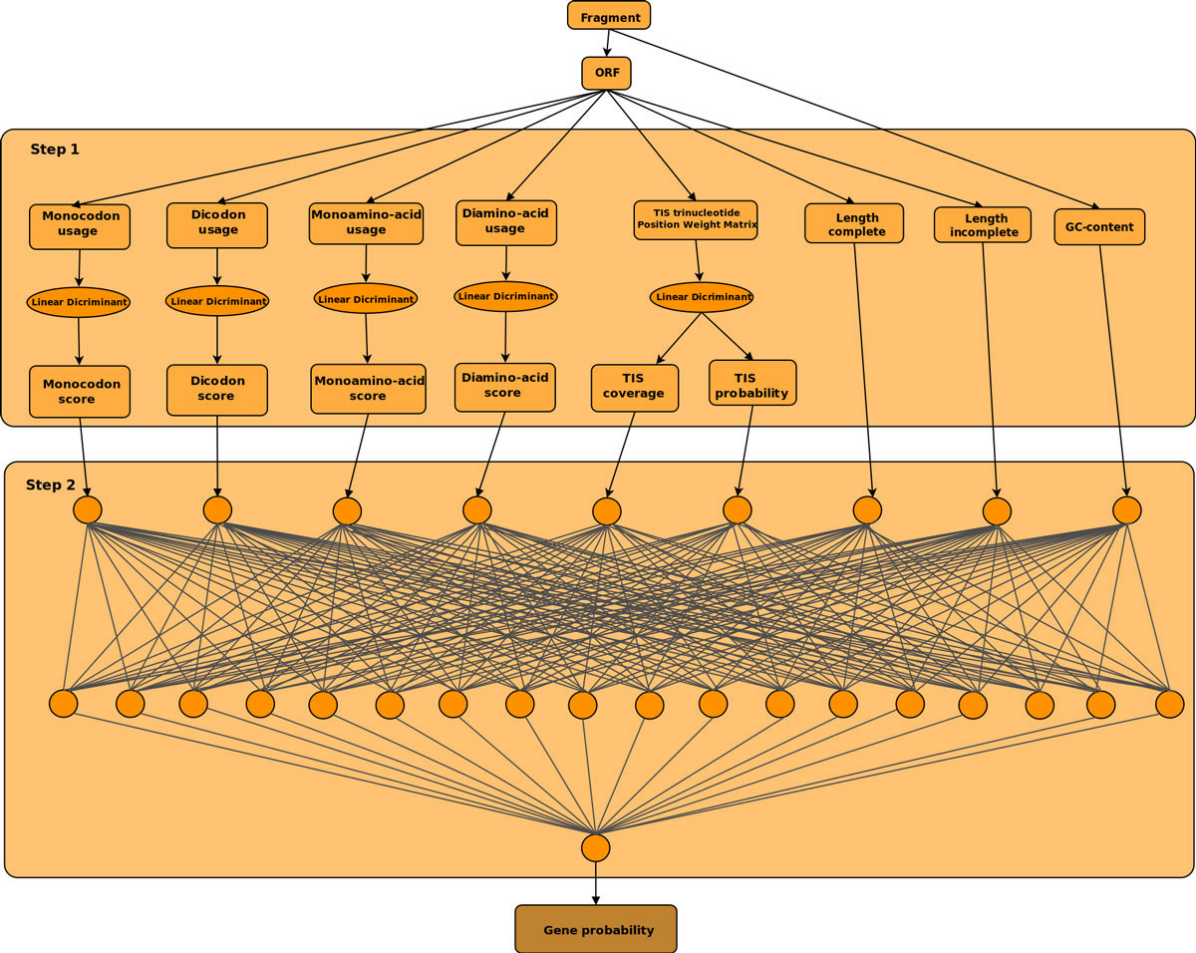
**MGC's scoring scheme**. **The figure illustrates MGC's scoring scheme**. The first steps computes six features from the ORF based on the corresponding linear discriminant. Three additional features are computed directly from the ORF. The neural network model from the corresponding GC range is used to combine features from the previous step in order to compute a final gene probability.

Several linear discriminants are trained based on GC-content ranges. First the training data is split into GC ranges which are defined so that the number of training sequences in all these ranges is the same. For example, we first split the GC spectrum into ranges where each partition contains 10% of the sequences in the training data, then we use the data from each range to create all the necessary discriminants to compute the features. Step 1 in Figure 2 illustrates the linear discriminant stage of MGC for a particular GC range and shows all nine features used in the second stage of the MGC algorithm.

For each GC range we obtain a model using features computed from all the sequences in the training dataset that have GC-content within the GC range. The same GC ranges used to compute the linear discriminants are used to build the neural network models. Different partitionings by GC-content are used to study the effect of the GC range size on the performance of MGC. In this paper we investigate the outcome of MGC models trained by partioning the training data into 10%, 5% and 2.5% ranges. For the remaining of this paper, we refer to these ranges as the 10%, 5% and 2.5% GC ranges.

Once the models are trained, all possible complete and incomplete ORFs are extracted from the input fragment and their corresponding features are extracted using the same linear discriminant step used for training. Based on the GC-content of the fragment, the corresponding neural network model is used to score the ORF. The output of the neural network is the approximation of the posterior probability that the ORF is coding. Step 2 in Figure 2 illustrates the neural network model. Once all input ORFs are scored by the neural networks, the same greedy algorithm used by Orphelia is deployed to resolve the overlap between all candidate ORFs that have a probability greater than 0.5. Given the candidate list  for a particular fragment containing all ORFs *i *with probability *P_i _>*0.5, Algorithm 1 describes the selection scheme used to generate the final list  of genes. The maximum allowed overlap is *o_max _*= 60 *bp *which is the minimal gene length considered for prediction. A more reasonable overlap would be 45 bp which is believed to be the maximum overlap for bacterial genes. We use the same overlap used by Orphelia for comparison reason. However, the overlap is a variable that the user can change.

**Algorithm 1 **The final candidate selection

    **while ** is nonempty **do**

        Find *i_max _*= argmax*_i _P_i _*with respect to all ORFs i in 

        Move ORF *i_max _*from  to 

        Remove all the ORFs in  that overlap with ORF *i_max _*by more than *o_max_*

    **end while**

### Features

In order to train the models in MGC we use the nine features (monoamino-acid discriminant, diamino-acid discriminant, monocodon discriminant, dicodon discriminant, two TIS features, two length features and the GC-content). Similarly to standard discriminant codon features, the amino discriminant features are derived from amino acid usage. The monoamino-acid usage is based on the 21 (20 amino-acids plus "STOP") amino-acid frequencies that represent the occurrences of successive single amino-acids in the training sequences while the diamino-acid usage is derived from the 21^2 ^diamino-acid frequencies which represent the occurrences of successive half-overlapping amino acid tuples in the training sequences. Linear discriminant analysis based on the monoamino and diamino-acid usage is then used to reduce this high dimensional space to two features. The linear discriminants **w***_MA _*and **w***_DA _*for the amino-acid features are described by the following equations:

(1) $$w_{MA}={\left(X_{MA}X_{MA}^{T}+\lambda nI\right)}^{-1}X_{MA}y_{M}$$

(2) $$w_{DA}={\left(X_{DA}X_{DA}^{T}+\lambda nI\right)}^{-1}X_{DA}y_{D}$$

Where **X***_MA _*and **X***_DA _*represent the monoamino and diamino-acid usage respectively, λ is the regularization parameter and **y***_M _*and **y***_D _*represent the sequence labels for the data points in **X***_MA _*and **X***_DA _*respectively ($y_{M}^{i}\in \left\{-1,1\right\}$ represents whether sequence *i *is a positive example $\left(y_{M}^{i}=1\right)$ or a negative example $\left(y_{M}^{i}=-1\right)$). The linear discriminants for codon features are computed similarly. The monoamino-acid and diamino-acid features are then obtained simply as *x *= **w***_MA _· ***x***_MA _*and *x *= **w***_DA _· ***x***_DA _*respectively.

### Neural networks

The resulting nine features for all the training examples in each GC range are combined in a non-linear fashion using a neural network. The output of each network is the posterior probability of an ORF encoding a protein. We use a standard multilayer perceptron to train the MGC models. This is similar to Orphelia [1] with the exception that we have two more features, and we are training models that are GC range specific. For each GC range we obtain a model using features computed from all the sequences in the training dataset that have GC-content within the GC range. The same GC ranges used to compute the linear discriminants are used to build the neural network models. Different splits by GC-content were used to study the effect of the GC range size on the performance of MGC. In this paper, the MGC models were trained using the 10%, 5% and 2.5% ranges.

The neural network used by Orphelia [18] consists of standard multilayer perceptrons with one layer of k hidden nodes and a single logistic output function is used to train the neural network model. While the classification is setup as a binary classification with labels *y_i _*= 1 for coding and *y_i _*= 0 for noncoding, the output of the neural network is considered an approximation of the posterior probability of the coding class which is used in the final step to select the final ORFs. The k hidden activations *z_i _*for a given input feature vector **x **are:

(3) $$z_{i}=\text{tanh}\left(w_{I}^{i}\cdot x+b_{I}^{i}\right).$$

where $w_{I}^{i}$ are input weight vectors and $b_{I}^{i}$ are the bias parameters.

The prediction function based on weight vector **w***_o _*and bias *b_o _*is

(4) $$g\left(z\right)=\frac{1}{1+\text{exp}\;\left(-w_{o}\cdot z-b_{o}\right)}.$$

where z is a vector containing all the *z_i _*vectors.

The output of the trained network $f\left(x_{i};\theta \right)\;\forall i\in \left(1..N\right)$ is computed by minimizing the objective function *E*(*θ*) in equation 5 where *x_i _*represent the training examples, *N *is the number of training examples, the weight and bias parameters are referred to by the vector *θ *and the matrix **A **contains the regularization parameters.

(5) $$E\left(\theta \right)=\overset{N}{\underset{i=1}{\sum }}{\left(f\left(x_{i};\theta \right)-y_{i}\right)}^{2}+\theta^{T}A\theta .$$

The regularization matrix **A **= *diag*(*a*1, ..., *a*1, *a*2, ..., *a*2, *a*3, ..., *a*3, *a*4) requires four strictly positive hyperparameters *a*_1_, *a*_2_, *a*_3_, *a*_4 _for separate scaling of the parameters $w_{I}^{i}$, $b_{I}^{i}$, **w***_o_*, *b_o_*. Hoff et al. [18] use the evidence framework for the adaptation of hyperparameters. This framework is introduced by MacKay [19] and is based on a Gaussian approximation of the posterior distribution of network weights. This evidence-based adaptation of the hyperparameters is incorporated into the network training and uses the same training points.

In order to minimize the objective function in equation 5, a scaled conjugate gradient scheme is used as implemented in the NETLAB toolbox [20]. The hyperparameters are all initially set to 0.001 and the weight and bias parameters are randomly initialized based on a standard normal distribution. The training scheme is iterated 50 times where each iteration consists of 50 gradient steps followed by two hyperparameter adaptation steps.

For example if we consider the 10% GC ranges, MGC computes 10 models using the training sequences from each GC range. Let *θ_j_*, where *j *∈ 1..10, denote the resulting neural network model for a given GC range *j*. Training the model *θ_j _*is similar to training the single model *θ *as described above and using only the training examples that have GC-content within the GC range *j*. The network output for a given test sample **x***_i _*is computed as *f*(**x***_i_*; *θ_j_*) = *P_i_*, where the GC-content of the fragment that contains **x***_i _*is within the GC range *j*.

## Results and discussion

### Performance measures

The performance of MGC is measured using the sensitivity and specificity measures which evaluate the capability of detecting annotated genes and the reliability of the gene predictions respectively. The performance measures are computed for predicted genes in fragments with length 700 bp from 10 random replications of 10 bacterial and 3 archaeal genomes based on their GenBank [17] annotations.

In order to measure the performance of the neural network Hoff et al. [18] use the sensitivity and specificity measures in equations 6 and 7 to measure the capability of detecting annotated genes and the reliability of gene predictions respectively. *TP_gene _*is the number of ORFs that match at least 60 bp on an annotated gene in the same reading-frame, while *FN_gene _*is the number of overlooked genes and *FP_gene _*refers to the number of predicted ORFs that do not match the annotation. For comparison reasons we follow the same use of the positive likelihood score as a measure of specificity, this score does not take into account the number of true negatives and is used by metagenomic gene finders such as Orphelia, FragGeneScan, and MetaGene.

(6) $$Sens=\frac{TP_{gene}}{TP_{gene}+FN_{gene}}.$$

(7) $$Spec=\frac{TP_{gene}}{TP_{gene}+FP_{gene}}.$$

The harmonic mean is also used to provide a composite of the sensitivity and specificity:

(8) $$HarmonicMean=\frac{2\times Sens\times Spec}{Sens+Spec}.$$

The accuracy of TIS was measured using the TIS correctness measure in equation 9. This measure is used because the traditional sensitivity and specificity measures are not suitable for measuring TIS prediction performance since they measure the performance of the gene prediction rather than the TIS accuracy.

*TP_TIS _*refers to the correctly predicted TIS within a subset of *TP_gene _*predictions that have an annotated TIS within the fragment, the latter is referred to as TP_gene*_.

(9) $$TIScorrectness=\frac{TP_{TIS}}{TPgene^{*}}.$$

## Results

Table 1 shows the sensitivity, specificity and harmonic mean scores of MGC predictions based on models built from 10%, 5% and 2.5% GC ranges respectively. The harmonic mean score is a composite measure of sensitivity and specificity [18]. Models built from the 10% GC ranges have an average harmonic mean of 91.44% with an average standard deviation of 0.15%. The 5% and 2.5% GC ranges have a slightly lower harmonic mean than the 10% ranges. Their average harmonic means are 91.32% and 90.61% respectively, and the average standard deviations for both ranges are 0.15%. Based on these results we select the 10% range for comparison with existing methods. Both Orphelia and FragGeneScan outperform MetaGene [7,18]. Consequently, we compare MGC's performance to that of Orphelia and FragGeneScan.

**Table 1:** MGC performance by GC ranges.

Model Ranges	10% Ranges			5% Ranges			2.5% Ranges		
**Genomes**	**Sp**	**Sn**	**H.M**	**Sp**	**Sn**	**H.M**	**Sp**	**Sn**	**H.M**

*M. jannaschii*	97.19±0.12	92.63±0.19	94.85±0.13	97.240.14±	92.78±0.18	94.85±0.11	97.10±0.10	92.67±0.18	94.84±0.12

*A. fulgidus*	95.04±0.14	83.87±0.18	89.11±0.11	94.950.16±	83.75±0.21	89.00±0.15	94.30±0.18	84.41±0.22	89.08±0.14

*B. subtilis*	96.68±0.13	88.06±0.17	92.17±0.12	96.63±0.12	88.03±0.18	92.13±0.14	96.20±0.09	87.81±0.13	91.82±0.09

*B. aphidicola*	98.01±0.19	91.11±0.37	94.43±0.23	98.00±0.17	90.82±0.39	94.27±0.25	97.89±0.22	90.54±0.34	94.07±0.22

*W. endosymbiont*	88.25±0.35	87.85±0.17	88.05±0.24	87.940.29±	87.99±0.24	87.97±0.23	87.39±0.26	88.15±0.18	87.77±0.21

*N. pharaonis*	95.28±0.12	85.79±0.20	90.29±0.14	94.91±0.11	85.29±0.30	89.84±0.19	94.41±0.13	81.60±0.26	87.54±0.17

*E. coli*	96.47±0.08	87.73±0.16	91.92±0.08	96.44±0.09	87.65±0.14	91.84±0.07	95.66±0.09	86.64±0.14	90.93±0.09

*H. pylori*	97.77±0.14	89.70±0.22	93.56±0.17	97.81±0.10	89.59±0.19	93.52±0.14	97.73±0.09	88.96±0.23	93.14±0.16

*P. aeruginosa*	96.16±0.09	91.70±0.11	93.88±0.08	95.93±0.09	91.53±0.09	93.67±0.07	95.72±0.08	89.17±0.13	92.33±0.09

*C. tepidum*	93.42±0.14	79.08±0.24	85.65±0.18	93.33±0.15	79.04±0.19	85.59±0.14	92.37±0.14	77.94±0.18	84.54±0.14

*B. pseudomallei*	94.79±0.13	87.84±0.25	91.18±0.18	94.46±0.12	87.59±0.24	90.90±0.16	93.99±0.13	85.86±0.19	89.74±0.15

*C. jeikeium*	96.13±0.11	87.70±0.23	91.72±0.17	95.81±0.08	87.53±0.23	91.48±0.15	85.02±0.14	95.56±0.26	89.98±0.20

*P. marinus*	97.71±0.11	87.92±0.20	92.55±0.12	97.57±0.13	88.28±0.20	92.69±0.14	88.04±0.14	97.47±0.24	92.51±0.13

**Average**	95.51	87.76	91.44	95.37	87.67	91.32	94.97	86.70	90.61

**Average S.D**.	0.14	0.20	0.15	0.14	0.21	0.15	0.14	0.21	0.15

This table presents the gene prediction performance of MGC using the 10%, 5% and 2.5% models. Sensitivity (Sn), Specificity (Sp) and Harmonic Mean (H.M) scores are measured on 700 bp randomly excited fragments from each test genome to 5-fold coverage and repeated 10 times.

Table 2 shows a comparison between MGC, Orphelia, and FragGeneScan using the testing dataset described earlier in this paper. The MGC algorithm is run using the 10% GC range models. The results show that both MGC and Orphelia achieve better performance than FragGeneScan across all accuracy scores. Therefore we will focus on comparing MGC and Orphelia. The average harmonic mean for MGC is 91.44% and the average standard standard deviation of the harmonic mean is 0.15%, while the average harmonic mean for Orphelia is 81.73% with an average standard deviation of 0.2%. We observe that MGC's improvement over Orphelia is both in sensitivity (8.87% on average) and in specificity (10.68% on average) measures. Orphelia was originally compared to MetaGene in Hoff et. al [1], where it was shown that Orphelia has an average of 4.6% specificity gain and a 3.8% sensitivity loss compared to MetaGene based on the same test species used in our comparison. However, the overall performance measured by the harmonic mean was very similar between Orphelia and MetaGene. In the case of MGC, both aggregate performance measures have improved and the harmonic mean shows an improvement of 9.71% on average over the results of Orphelia.

**Table 2:** MGC versus Orphelia and FragGeneScan.

Methods	MGC			Orphelia			FragGeneScan		
**Genomes**	**Sp**	**Sn**	**H.M**	**Sp**	**Sn**	**H.M**	**Sp**	**Sn**	**H.M**

*M. jannaschii*	**97.19**±0.12	**92.63**±0.19	**94.85**±0.13	95.20±0.17	90.46±0.16	92.77±0.14	76.03±0.22	90.35±0.33	82.57±0.19

*A. fulgidus*	**95.04**±0.14	**84.13**±0.23	**89.31**±0.15	88.57±0.21	80.58±0.17	84.38±0.16	52.58±0.3	75.86±0.31	62.11±0.29

*B. subtilis*	**96.68**±0.13	**88.06**±0.17	**92.17**±0.12	88.91±0.12	83.45±0.11	86.10±0.09	66.47±0.25	78.98±0.22	72.19±0.23

*B. aphidicola*	**98.01**±0.19	**91.11**±0.37	**94.43**±0.23	95.54±0.28	89.40±0.33	92.37±0.22	80.91±0.56	92.2±0.32	86.19±0.34

*W. endosymbiont*	**88.25**±0.35	**87.85**±0.17	**88.05**±0.24	86.24±0.39	83.79±0.31	84.99±0.27	71.44±0.49	71.24±0.54	71.34±0.45

*N. pharaonis*	**95.28**±0.12	**85.79**±0.20	**90.29**±0.14	75.99±0.34	68.74±0.34	72.17±0.33	52.89±0.37	63.62±0.34	57.76±0.36

*E. coli*	**96.47**±0.08	**87.73**±0.16	**91.92**±0.08	85.99±0.18	80.79±0.16	83.31±0.16	62.57±0.2	74.93±0.19	68.19±0.15

*H. pylori*	**97.77**±0.14	**89.70**±0.22	**93.56**±0.17	94.17±0.20	88.99±0.22	91.50±0.20	72.76±0.35	87.54±0.39	79.47±0.32

*P. aeruginosa*	**96.16**±0.09	**91.70**±0.11	**93.88**±0.08	71.21±0.20	68.40±0.18	69.78±0.19	56.17±0.3	63.46±0.3	59.59±0.29

*C. tepidum*	**93.42**±0.14	**79.08**±0.24	**85.65**±0.18	77.51±0.22	66.95±0.23	71.85±0.21	50.87±0.36	65.59±0.22	57.3±0.29

*B. pseudomallei*	**94.79**±0.13	**87.84**±0.25	**91.18**±0.18	69.54±0.31	64.79±0.22	67.08±0.26	51.34±0.2	55.69±0.27	53.42±0.22

*C. jeikeium*	**96.13**±0.11	**87.70**±0.23	**91.72**±0.17	79.52±0.22	74.23±0.23	76.79±0.22	65.41±0.28	72.78±0.3	68.9±0.26

*P. marinus*	**97.71**±0.11	**87.92**±0.20	**92.55**±0.12	94.41±0.20	84.98±0.24	89.45±0.20	75.48±0.4	88.49±0.32	81.47±0.33

**Average**	**95.51**	**87.76**	**91.44**	84.83	78.89	81.73	64.22	75.44	69.27

**Average S.D**.	**0.14**	**0.20**	**0.15**	0.23	0.22	0.20	0.33	0.31	0.29

This table compares the prediction performance of MGC, Orphelia [1] and FragGeneScan [7]. Sensitivity (Sn), Specificity (Sp) and Harmonic Mean (H.M) scores are derived identically to Table 1.

Predicting the correct TIS is very important and challenging in conventional as well as metagenomic gene finding. This is crucial to the subsequent experimental steps in the metagenomic pipeline. MGC employs linear discriminant TIS-models in order to identify the correct TIS. The accuracy of this prediction can be measured using the TIS correctness score as described in the previous section. Table 3 shows a comparison of TIS correctness scores between MGC and Orphelia.

**Table 3:** TIS accuracy comparison between MGC and Orphelia.

	MGC	Orphelia
**Genomes**	**TIScorrectness**	**TIScorrectness**

*M. jannaschii*	64.12 ± 0.84	51.03 ± 0.85

*A. fulgidus*	66.26 ± 0.39	51.10 ± 0.60

*B. subtilis*	65.47 ± 0.22	58.85 ± 0.25

*B. aphidicola*	84.15 ± 0.70	65.30 ± 1.44

*W. endosymbiont*	72.06 ± 0.81	63.41 ± 0.94

*N. pharaonis*	71.46 ± 0.35	59.43 ± 0.63

*E. coli*	72.74 ± 0.27	64.24 ± 0.35

*H. pylori*	68.55 ± 0.71	60.37 ± 0.71

*P. aeruginosa*	68.86 ± 0.42	61.09 ± 0.35

*C. tepidum*	69.49 ± 0.65	53.93 ± 0.71

*B. pseudomallei*	67.85 ± 0.46	56.11 ± 0.69

*C. jeikeium*	71.33 ± 0.69	60.29 ± 0.57

*P. marinus*	71.18 ± 0.37	68.32 ± 0.38

**Average**	70.89	59.50

**Average S.D**.	0.53	0.68

TIScorrectness scores are measured on 700 bp randomly excited fragments from each test genome to 5-fold coverage and repeated 10 times.

TIS correctness measure is computed from a subset of the predicted genes with an annotated TIS. Direct comparison of the two methods based on this measure is difficult since they predict a different number of genes. Nonetheless, we notice that the improvement of the TIS correctness is comparable to that of the sensitivity and specificity measures. Specifically, we observe that the average TIS correctness of our algorithm is 11.39% higher than that of Orphelia.

## Discussion

The results show the improvement of MGC in performance over that of Orphelia. We hypothesized that learning separate models for several pre-defined GC-content regions as opposed to the single model approach used by Orphelia would improve the performance of the neural network. The current results support this hypothesis. The 5% GC range models exhibit an improvement around 1% on average than that of the 2.5% GC range models. The 10% GC range models also exhibit a slight improvement over the 5% GC range models. However this result is within the standard deviation. Thus, there is no need to investigate models for smaller GC ranges to prove the benefit of having multiple models versus a single model. However, it would be useful to compute other models based on larger GC ranges in order to investigate and find better partitions of the GC spectrum.

MGC outperforms Orphelia in TIS prediction accuracy. Evaluating TIS recognition is hampered by the fact that we must rely on published annotations, many of which are generated automatically and have not been fully verified. This is a well recognized problem in traditional gene annotation.

The Orphelia algorithm was tested on different fragment sizes by building models for fragments ranging from 200 bp to 500 bp with increments of 20 bp. Hoff et al. recommend using the 700 bp model for all fragments greater than 300 bp, while fragments ranging from 200 bp to 300 bp should be run using the 300 bp model [1]. According to a recent metagenomics survey by Thomas et al. [21] the 454/Roche and the Illumina/Solexa systems are the most commonly used systems. While the Illumina/Solexa system produce shorter reads, the average read length for 454/Roche technology ranges between 600 and 800 bp [21]. MGC's 700 bp models are sufficient for longer reads such as 454/Roche reads. We are currently developing 300 bp models in order to handle shorter reads such as those from the llumina/Solexa system.

## Conclusion

In this paper we show that learning separate models for several pre-defined GC-content regions as opposed to the single model approach used by Orphelia lead to an improvement of performance. We also show that the amino-acid usage helps to improve the overall accuracy of the gene finder. In the future, we plan to evaluate models based on different GC ranges. We also plan to use ensemble techniques to combine the ORF probabilities from overlapping models in order to improve the predictions of MGC. This hypothesis is based on the empirical observation of Hansen and Krogh [22] that the error of an ensemble is the average error of each ensemble members minus a measure of the disagreement between each members. This suggests that the ensemble is always better than the individual average performance.

In our experiments, we have used simulated data derived from fully sequenced genomes. We plan to study the effect of sequencing errors on the prediction performance by simulating data with different error rates. Three types of errors can occur in all sequencing techniques: substitution, insertion, and deletion of one or more nucleotides during the reading process. Since we rely on codon and amino acid features to predict genes, any insertion or deletion will shift the frame of the sequence and thus alter the codon and amino acid compositions. In addition to evaluating MGC's prediction ability on sequences with these types of errors. We need to develop a way to compensate for the frame shifts, otherwise we will not be able to classify erroneous fragments. FragGeneScan currently shows the best performance for reads with errors. Once we address error modeling in MGC, we plan to compare our results with those of FragGeneScan.

## Competing interests

The authors declare that they have no competing interests.

## Authors' contributions

AE and JR conceived of the project. AE designed and implemented the work. JR helped in the design and provided expert input. Both authors read and approved the final manuscript.

## Declarations

Publication of this article was funded by the authors.
